# Fibril Growth Kinetics Reveal a Region of β_2_-microglobulin Important for Nucleation and Elongation of Aggregation

**DOI:** 10.1016/j.jmb.2008.01.092

**Published:** 2008-04-18

**Authors:** Geoffrey W. Platt, Katy E. Routledge, Steve W. Homans, Sheena E. Radford

**Affiliations:** Astbury Centre for Structural Molecular Biology and Institute of Molecular and Cellular Biology, University of Leeds, Leeds LS2 9JT, UK

**Keywords:** β_2_m, β_2_-microglobulin, ThT, thioflavin-T, HSQC, heteronuclear single quantum coherence, MHC, major histocompatibility complex, EM, electron microscopy, β_2_-microglobulin, amyloid, NMR relaxation, unfolded state, aggregation kinetics

## Abstract

Amyloid is a highly ordered form of aggregate comprising long, straight and unbranched proteinaceous fibrils that are formed with characteristic nucleation-dependent kinetics *in vitro*. Currently, the structural molecular mechanism of fibril nucleation and elongation is poorly understood. Here, we investigate the role of the sequence and structure of the initial monomeric precursor in determining the rates of nucleation and elongation of human β_2_-microglobulin (β_2_m). We describe the kinetics of seeded and spontaneous (unseeded) fibril growth of wild-type β_2_m and 12 variants at pH 2.5, targeting specifically an aromatic-rich region of the polypeptide chain (residues 62–70) that has been predicted to be highly amyloidogenic. The results reveal the importance of aromatic residues in this part of the β_2_m sequence in fibril formation under the conditions explored and show that this region of the polypeptide chain is involved in both the nucleation and the elongation phases of fibril formation. Structural analysis of the conformational properties of the unfolded monomer for each variant using NMR relaxation methods revealed that all variants contain significant non-random structure involving two hydrophobic clusters comprising regions 29–51 and 58–79, the extent of which is critically dependent on the sequence. No direct correlation was observed, however, between the extent of non-random structure in the unfolded state and the rates of fibril nucleation and elongation, suggesting that the early stages of aggregation involve significant conformational changes from the initial unfolded state. Together, the data suggest a model for β_2_m amyloid formation in which structurally specific interactions involving the highly hydrophobic and aromatic-rich region comprising residues 62–70 provide a complementary interface that is key to the generation of amyloid fibrils for this protein at acidic pH.

## Introduction

More than 20 disorders, including Alzheimer's and Creutzfeldt–Jacob diseases, are known to be linked with the deposition of insoluble amyloid aggregates.^1^ Amyloid is a highly ordered form of aggregate, comprising long, straight and unbranched proteinaceous fibrils that are thought to be formed as a result of protein misfolding.^2^ Such fibrils have been shown to have a common cross-β structure in which polypeptide β-strands are arranged in a perpendicular fashion with respect to the fibril long axis.^3,4^ It has been observed that many proteins, including some that are not involved in any known disease, can also form amyloid-like fibrils *in vitro* under appropriate conditions.^2^ This has led to the hypothesis that the ability to form amyloid fibrils is a generic feature of polypeptide chains, although the propensity for this can be greatly affected by the primary sequence.^5^ The formation of amyloid-like fibrils *in vitro* occurs with characteristic kinetics wherein a lag phase, involving the formation of a high-energy nucleus, precedes a period of exponential growth that corresponds to fibril elongation.^6^ Despite an increasing number of studies examining the influence of changes in the amino acid sequence on the propensity of different peptides and proteins to form amyloid fibrils,^7^ the structural molecular events that occur during nucleation and elongation remain unresolved.

Here, we describe a series of experiments that aims to elucidate the influence of the primary sequence and the structural properties of the initial unfolded monomer on the determination of the rates of nucleation and elongation of the amyloidogenic protein, human β_2_-microglobulin (β_2_m). β_2_m is the major protein component of amyloid plaques found in patients suffering from dialysis-related amyloidosis (reviewed by Radford *et al.*^8^). Native β_2_m contains 100 residues (∼ 12 kDa) and has a seven-stranded β-sandwich fold stabilised by a single disulphide bond linking residues 25 and 80 that is typical of the immunoglobulin superfamily.^9^ This bond has been shown to remain intact in fibrils *ex vivo* and to be required for the formation of long, straight amyloid-like fibrils *in vitro*.^10–13^ A number of studies have shown that fibril assembly from β_2_m occurs rapidly under acidic conditions *in vitro*.^14–16^ Thus, at pH 2.5, β_2_m spontaneously and rapidly assembles in the absence of fibrillar seeds to form amyloid-like fibrils with > 90% yield, with morphological and tinctorial properties characteristic of amyloid formed *in vivo*.^17^ The fibrils formed under these conditions are long, straight and twisted; bind the dye thioflavin-T (ThT); display apple-green birefringence in the presence of Congo red when viewed under cross-polarised light; and bind serum amyloid protein, glycosaminoglycans, amyloid-specific antibodies and other factors known to be associated with amyloid *in vivo*.^14,17,18^ These fibrils also give rise to an X-ray fibre diffraction pattern consistent with a cross-β fold.^17^ The growth kinetics under these conditions are also typical of the formation of amyloid fibrils, wherein an elongation phase is preceded by a lag phase, which can be removed by addition of fibrillar seeds.^19^ β_2_m also forms fibrils with a short, curvilinear morphology at acidic pH in buffers at high ionic strength.^14^ These fibrils form rapidly through a non-nucleated mechanism, on a pathway parallel to that which results in the formation of long, straight fibrils by nucleated growth.^20^

Previous NMR studies have indicated that β_2_m is highly unfolded in the low-pH conditions commonly used to initiate the nucleated growth of fibrils *in vitro*.^11,21^ Akin to the acid-denatured states of other proteins, *T*_2_ relaxation experiments have indicated that β_2_m contains significant non-random structure at pH 2.5, involving two hydrophobic clusters that are stabilised by the disulphide bridge connecting Cys25 and Cys80.^21^ These hydrophobic clusters involve residues 29–51 and 58–79, the latter of which is highly enriched in aromatic amino acids and has previously been highlighted as one of the most aggregation-prone regions of the sequence based on studies of synthetic peptides spanning all seven β-strands in the native state,^22^ as well as by several prediction algorithms.^23–25^

To investigate the role of residues 62–70 in the formation of amyloid fibrils from full-length β_2_m *in vitro*, and particularly the influence of the highly aromatic nature of this part of the sequence in fibril growth, we have made a number of substitutions focussed on this region of the polypeptide sequence. By developing assays capable of reliable analysis of the rates of nucleation and elongation, we determine the role of individual residues in this region of the polypeptide sequence in these fibril formation processes. The results demonstrate a key role of aromatic residues in this part of the protein in both fibril nucleation and elongation. By comparing the rates obtained with those predicted by algorithms based on the simple physicochemical properties of the polypeptide chain, and by taking into consideration the role of the structure of the initial unfolded state, we suggest a mechanism for β_2_m amyloid assembly involving a partially structured intermediate in which aromatic residues in the region 62–70 form at least one key interacting surface.

## Results

### Role of individual residues in residual structure in the acid-unfolded state

**Fig. 1:**
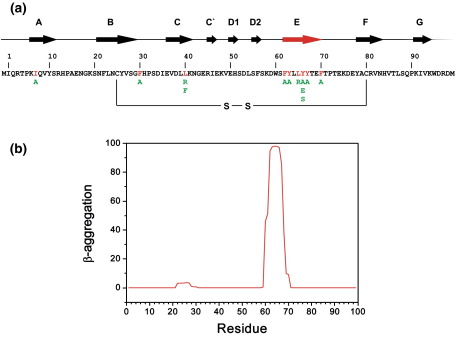
(a) Sequence of human β_2_m, with native secondary structural elements indicated. The position of the disulphide bond in the sequence is highlighted. Amino acids that were targeted in this study are highlighted in red, and the alterations made are show in green. (b) TANGO prediction for β-aggregation of the β_2_m sequence,^23^ indicating that the region corresponding to native β-strand E (residues 62–70) is highly aggregation-prone.

One of the most striking characteristics of the region comprising residues 62–70 in β_2_m is its high content of aromatic amino acids. Thus, of the nine residues that comprise this region, five are aromatic (Fig. 1a). This feature possibly rationalises the predicted high amyloidogenicity of this region of the protein^23–27^ (Fig. 1b) and the ability of synthetic peptides encompassing this region to rapidly form amyloid fibrils *in vitro*.^22,28^ To ascertain whether the aromatic character of residues 62–70 is important in the aggregation process of intact β_2_m under acidic conditions *in vitro*, eight variants were created in this region of the protein, and their effect on the kinetics of aggregation was monitored using the fluorescence of ThT as a probe of fibril formation. Modifications involved substituting aromatic residues with aliphatic side chains (F62A, Y66A, Y67A and F70A; Fig. 1a) to probe the effect of aromaticity on amyloid formation, as well as the introduction of a single charged amino acid in a central position in this region (L65R)—a feature predicted to be unfavourable for aggregation.^5,29,30^ In addition, Tyr66 was substituted with three different residues (Y66A, Y66E and Y66S) to determine whether changes in the kinetics of fibril formation could be related to the chemical properties of the introduced side chain.^23,26,30^ Finally, three aromatic residues were removed simultaneously from the variant F62AY63AY67A. This variant has been created hitherto and, most interestingly, shown to greatly disrupt residual structure in the acid-unfolded state,^21^ providing an opportunity to assess the role of structure in the acid-unfolded monomeric precursor in determining aggregation rates. Finally, to investigate whether any changes in aggregation kinetics observed are specific to residues 62–70 or merely reflect a general alteration in the solubility or hydrophobicity of the polypeptide chain, variants containing amino acid substitutions that decrease hydrophobicity (I7A), decrease or increase aromaticity (F30A and L40F) or introduce charge (L40R) were made in other regions of the protein (Fig. 1a).

**Fig. 2:**
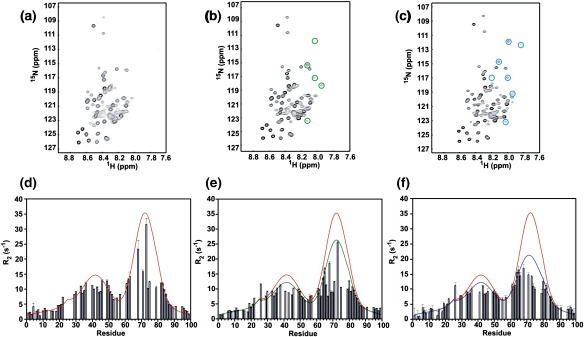
^1^H–^15^N HSQC spectra of (a) wild-type, (b) F62A and (c) L65R β_2_m. In (b) and (c), the peaks circled in green and blue, respectively (many of which correspond to residues in the region 62–70), are not detectable in the spectrum of the wild-type protein. Relaxation rates (*R*_2_) for (d) wild-type, (e) F62A and (f) L65R β_2_m. The data in (d)–(f) have been fitted using a random coil model that contains two regions of non-random structure, in addition to the single disulphide bond (see Materials and Methods).^21^ The fit for wild-type β_2_m is also shown in red in (e) and (f) to facilitate comparison.

**Fig. 3:**
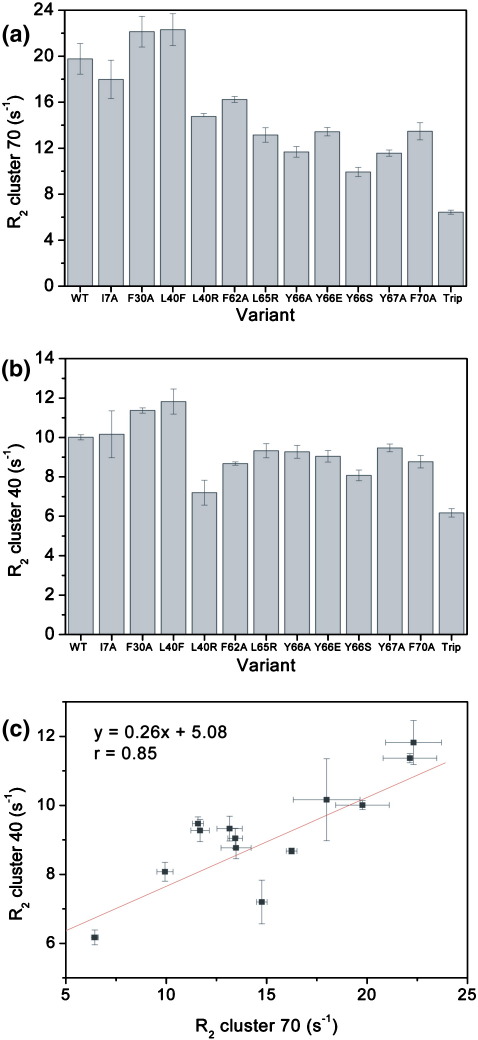
Average *R*_2_ rates for all of the variants studied here. ‘Trip’ refers to the triple mutant F62AY63AY67A. (a) *R*_2_ values for the hydrophobic cluster centred at residue 70 for each variant, obtained from average values for residues 61, 68, 73 and 75. (b) *R*_2_ values for the hydrophobic cluster centred at residue 40 for each variant (based on average *R*_2_ values for residues 33, 34, 41 and 43 that lie in this cluster). (c) Interaction between the two clusters demonstrated by plotting the average *R*_2_ values for each cluster in each variant against each other.

To determine the effect of the amino acid substitutions on the structure and dynamics of the unfolded state of β_2_m at pH 2.5 (the initial starting point of the aggregation reactions described below), ^1^H–^15^N heteronuclear single quantum coherence (HSQC) spectra of wild-type β_2_m and the 12 variants were acquired, and the relaxation properties of individual resonances were determined. The resulting spectra indicated that all 13 proteins are highly unfolded at pH 2.5, with the majority of residues giving rise to intense resonances with little chemical shift dispersion (Fig. 2a–c). Consistent with previous studies of the wild-type protein at this pH,^11,21^ measurement of the *R*_2_ relaxation rates revealed a number of resonances that are significantly broadened, whilst others are broadened beyond detection. These cluster into two regions of the sequence involving residues ∼ 29–51 and ∼ 58–79, consistent with these regions deviating from a random structure under these conditions (Fig. 2d–f). Akin to the properties of the wild-type protein (Fig. 2a and d),^21^ residues 62–70 in all of the variants (corresponding to β-strand E in native β_2_m; Fig. 1a) show the most broadened resonances, whilst the second group of residues (29–51) also displays significant broadening, consistent with the presence of two hydrophobic clusters in the acid-unfolded state. Importantly, substitution of residues in the region 62–70 not only affects the dynamics within its own cluster but also perturbs those of the residues in the cluster centred on residue 40, indicating that these two regions are mutually interacting (Fig. 3a–c). By contrast, Ile7, a residue that is highly dynamic in the acid-unfolded state and is not involved in the residual structure, has little effect on the dynamics of either cluster. Finally, and by contrast with the small to moderate changes in *R*_2_ observed when single point mutants are introduced into the region 62–70, reducing the aromatic content of this region substantially by replacing three aromatic residues with alanine simultaneously (F62AY63AY67A, hereinafter referred to as the triple mutant) results in the greatest reduction in *R*_2_ values in both clusters (Fig. 3a and b). Therefore, over the range of amino acid changes introduced, hydrophobic clustering involving residues 62–70 has been titrated from a behaviour similar to that of wild-type β_2_m (e.g., I7A), through proteins with partially disrupted clusters (as is the case with most of the single point variants in the region 62–70), to the triple mutant that is the most highly dynamic species of this set (Fig. 3a), enabling the importance of residual structure in this region for amyloid assembly to be explored.

### The role of aromatic interactions in fibril elongation

**Fig. 4:**
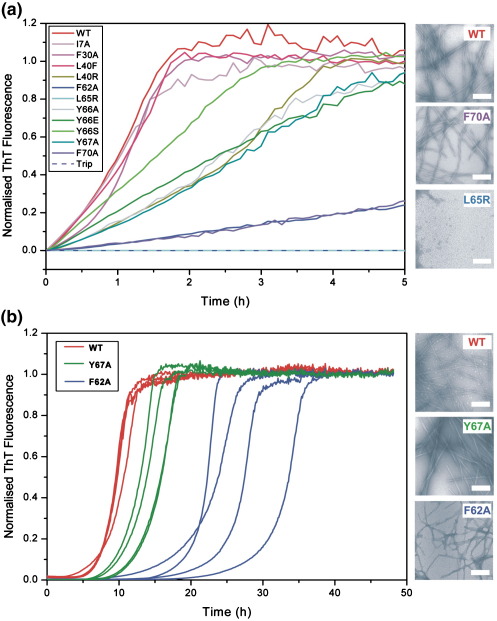
(a) Apparent elongation rates of β_2_m variants analysed using seeded fibril growth monitored by ThT fluorescence. Variants displaying apparent elongation kinetics similar to those of wild-type β_2_m are shown in red, those with intermediate apparent rates are shown in green and those with the slowest apparent rates are indicated in blue. Negative-stain EM images, taken after 24 h, for three example variants are shown on the right, with scale bars representing 200 nm. (b) Example ThT traces of spontaneous fibril formation for different variants of β_2_m. Data are presented for wild-type β_2_m (red), a variant with intermediate kinetics (Y67A; green) and a variant with a long lag time (F62A; blue). Negative-stain EM images for the fibrils formed from these three variants are shown on the right, with the scale bar representing 200 nm.

**Fig. 5:**
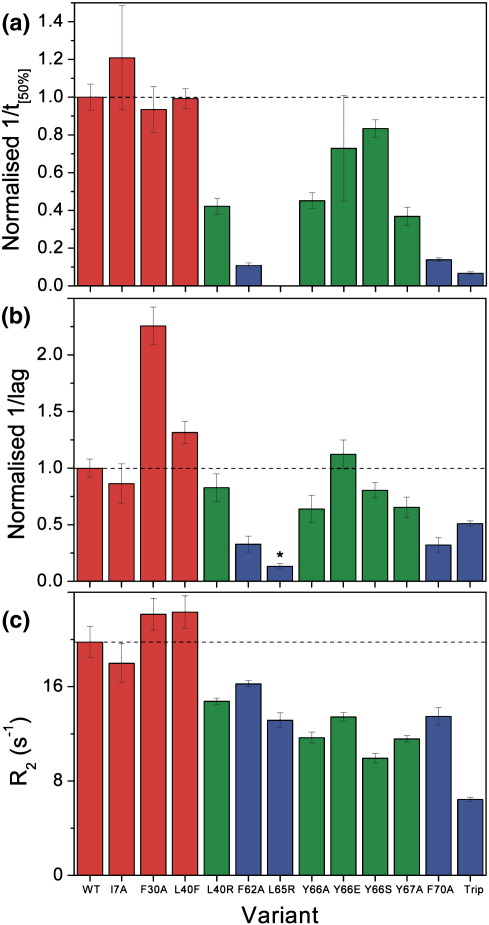
Comparison of fibril formation rates with structural properties of the initial unfolded state. (a) Relative apparent elongation rates (taken from 1/*t*_[50%]_ value) determined using seeded growth assays normalised to the data for wild-type β_2_m. The fastest rates are shown in red, intermediate rates are shown in green and the slowest rates are indicated in blue. (b) Normalised 1/lag times determined using spontaneous fibril growth experiments, coloured as in (a). (*) Not all of the reactions containing L65R resulted in fibril formation over the 75-h period studied. In these cases, the end point of the experiment (75 h) was taken as the lag time. The data shown, therefore, are an underestimate of the true lag time. (c) Rates of *T*_2_ relaxation for the hydrophobic cluster involving residues 62–70 calculated using an average of the values obtained for residues 61, 68, 73 and 75 for each variant. The bars are coloured according to the scheme used in (a) and (b).

The fibril elongation kinetics of each of the variants of β_2_m created was next monitored at pH 2.5 using ThT fluorescence. Experiments performed in parallel using intrinsic tryptophan fluorescence or fluorescence anisotropy confirmed that ThT fluorescence reflects the formation of amyloid-like fibrils, since similar rates of fibrillation are observed independent of the assay used (data not shown). Multiple samples of the monomeric state of each variant at pH 2.5 were set up simultaneously, added to fibril seeds formed from the wild-type protein (see Materials and Methods), and the apparent elongation kinetics of each sample was recorded using a 96-well plate reader at 25 °C. In each case, the apparent elongation rate obtained with the wild-type protein was recorded concurrently and used to normalise the data obtained for each variant. The resulting data (Fig. 4a) revealed that 10 of the 12 variants created are able to elongate wild-type fibril seeds under these conditions, resulting in fibrils that are morphologically indistinguishable from those formed from the wild-type protein, as expected for templated growth of proteins with high sequence identity.^31–33^ Most importantly, however, clear differences in the apparent rate of elongation were observed for different variants, dependent upon the site or the type of amino acid substitution made. Thus, some variants showed kinetics similar to that of wild-type β_2_m (e.g., I7A, F30A and L40F), whilst others resulted in apparent elongation rates that were substantially reduced (Figs. 4a and 5a). Most strikingly, all of the variants containing amino acid substitutions that remove an aromatic amino acid in the region 62–70 reduced the apparent rate of elongation significantly, whilst similar changes made elsewhere in the sequence resulted in little change in the rates (e.g., compare the data for F62A and F70A with the data for F30A; Figs. 4a and 5a). The data suggest, therefore, that aromatic residues in the region 62–70 form an interacting interface involved in the elongation of fibril seeds. Consistent with this view, the most dramatic effect of the 12 amino acid substitutions introduced was obtained with the variant L65R, where a single substitution in this region abolishes the ability of this protein to elongate wild-type seeds under the conditions employed (Figs. 4a and 5a). Importantly, the same type of substitution made elsewhere (L40R) had a much smaller effect, reinforcing the view that residues 62–70 play a key role in fibril elongation. Finally, whilst incubation of the triple mutant results in no increase in ThT fluorescence monitored over 5 h (Fig. 4a), a small but significant increase in ThT fluorescence was observed when the reaction was monitored over an extended time period. The rate observed, however, mirrors that observed in the absence of fibril seeds (see below), indicating that the removal of three aromatic residues from this region of the polypeptide chain results in a monomer that is unable to elongate wild-type seeds under these conditions.

### Mutational effects on the lag time

Nucleation of amyloid fibril growth is thought to involve the formation of an energetically unfavourable nucleus, which then permits rapid elongation and formation of fibrillar species.^6^ The development of assay conditions that permit the formation of fibrils with reproducible kinetics without any evidence for amorphous aggregation provides an opportunity to determine the role of individual residues in the nucleation process. To determine the role of aromatic residues in the region 62–70 in the nucleation of β_2_m fibril formation, the length of the lag time in the formation of amyloid-like fibrils of wild-type β_2_m and the 12 variants created was measured using an assay in which spontaneous assembly was monitored in the absence of fibril seeds (see Materials and Methods). Once again, multiple replicate samples of the wild-type protein and each variant were monitored in parallel, allowing the average lag time for each variant to be accurately measured and normalised to that of the wild-type protein. The results, shown in Figs. 4b and 5b, revealed that all of the variants are able to form long, straight fibrils that resemble those of the wild-type protein in these unseeded reactions, with lag times that vary substantially. For Y66E, however, fibrils of different morphologies were also observed. Thus, fibrils with the twisted rope-like structure observed for long, straight fibrils formed from wild-type β_2_m were found, mixed with a second fibril type consisting of flat twisted ribbons. All of the variants containing amino acid substitutions between residues 62 and 70 resulted in a significant increase in the lag time, except for Y66E. By contrast, the variants involving substitutions made outside this region, such as I7A and L40F, showed little change in the lag time, while F30A showed a reduction in the lag time. Again, the most dramatic effect was observed for the variant L65R. For this protein, 9 of the 12 replicates analysed did not result in the formation of long, straight fibrils within the 75-h timescale of this experiment, with fibril formation for these samples instead resulting in short curvilinear fibrils reminiscent of those formed from the wild-type protein at high ionic strengths after incubation for this time.^17,20^ Thus, the mutation L65R substantially disfavours nucleation, leaving open access to the competing non-nucleated assembly pathway,^20^ resulting in the formation of curvilinear fibrils under these conditions. Together, the data demonstrate that residues 62–70 play an important role in fibril nucleation, such that removing aromatic residues or adding a single charge in this region severely disrupts the ability of the sequence to nucleate fibril growth.

### Fibril nucleation and elongation involve similar interacting surfaces

**Fig. 6:**
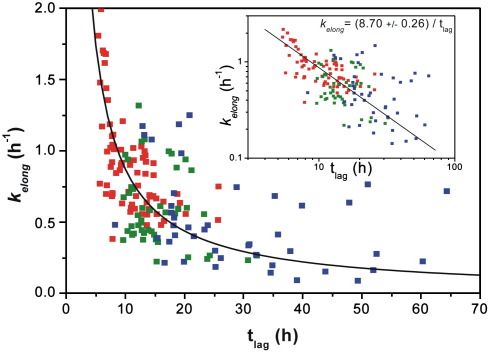
Scattergram of the apparent elongation rate *versus* the lag time of each individual spontaneous fibril growth experiment for all of the variants studied here. The inset shows a log–log plot of the same data with a linear fit, implying a common aggregation mechanism for all of the variants studied. The data points are coloured according to the same scheme as in Fig. 5.

Previous studies of amyloid formation *in vitro* using insulin, glucagon and different variants of Aβ(1–40) as model systems have shown that the length of the lag time and the apparent elongation rate are correlated.^34^ To determine whether this is the case for the variants of β_2_m created here, the apparent elongation rate and the lag time for each individual sample of each variant studied were plotted (Fig. 6). The results revealed a clear correlation between these two parameters, with reactions with the longest lag time showing the slowest apparent elongation rates and *vice versa*, implying a common mechanism of fibril formation for all of the variants studied (Fig. 6). Interestingly, although a correlation between the lag time and the apparent elongation rate is observed for β_2_m, quantitatively the dataset differs from that obtained previously for the aggregating peptide systems analysed by Fändrich, possibly reflecting the increased size and complexity of β_2_m relative to the systems studied to date.^34^ Alternatively, the difference could result from the different growth conditions used, such as quiescent *versus* agitation, with the latter being required to create fibrils from β_2_m on a measurable timescale without visible amorphous aggregation.

### Role of residual structure in acid-unfolded β_2_m in fibril growth

The structure in the acid-unfolded state of β_2_m (reflected by the *R*_2_ value for the 62–70 cluster) and the apparent rates of fibril nucleation and elongation are compared in Fig. 5. Whilst the relationship between these parameters is complex, qualitatively clear features emerge. Thus, all of the variants that have an average *R*_2_ value in the cluster 62–70 similar to that of wild-type β_2_m result in nucleation and apparent elongation rates akin to those of the wild-type protein (Fig. 5, red bars). For variants with reduced *R*_2_ values in this cluster, however, no clear correlation is observed between the *R*_2_, the lag time and the apparent elongation rate. For instance, Y66E and F70A display similar relaxation rates; however, the apparent rate of fibril elongation is far greater for Y66E. Similarly, F62A has one of the slowest rates of elongation and nucleation, but this substitution has only a moderate effect on the *R*_2_ values. Finally, whilst L65R has a dramatic effect on the ability of β_2_m to form amyloid fibrils, the *R*_2_ of this variant is only moderately perturbed. Similar results have been obtained previously for other proteins, wherein aggregation has been shown to be favoured by solution conditions that promote stable intermolecular interactions rather than inducing specific conformational properties in a partially unfolded state.^35,36^ Whilst the retention of a hydrophobic cluster in the region 62–70 in β_2_m is commensurate with rapid fibril growth, the precise effects of an amino acid substitution thus appear to depend critically on the type of substitution made and the location of the amino acid substituted, suggesting that the context of each amino acid in the polypeptide sequence and in the structure of the early aggregating species plays a critical role in determining the aggregation potential of each variant. Consistent with this conclusion, algorithms developed to predict aggregation potential or elongation rates, assuming that self-assembly involves an unstructured precursor in which every amino acid contributes equally and is dependent only upon its physicochemical properties, result in a poor prediction of the aggregation rates of the 12 variants created here (data not shown).^5,29,30^

## Discussion

### Importance of residues 62–70 in the aggregation of β_2_m

The kinetic data presented above show that of the 100 residues that comprise the sequence of β_2_m, residues 62–70 form at least one key interacting surface that is important in fibril formation. We show, using detailed kinetic analysis of fibril formation, that altering residues to reduce hydrophobicity or to introduce polar side chains into this hydrophobic region of the polypeptide chain increases the length of the lag time and decreases the apparent elongation rate of fibril formation, whilst identical amino acid substitutions made elsewhere in the protein have little effect on the rate of fibril formation. The importance of residues 62–70 in the fibrillogenesis of β_2_m has been suggested previously based on studies of synthetic peptides corresponding to each of the seven β-strands of native β_2_m, in which only the peptide containing this sequence, equivalent to β-strand E in the native protein, was shown to form fibrils in isolation,^22^ as well as by analysis of shorter six-residue amino acid sequences taken from this region.^28^ Strikingly, despite the identification of a stretch of only nine residues that is able to modulate or even ablate fibril formation dependent on its sequence, > 80 residues of this 100-residue protein are involved in the fibril core, as revealed by limited proteolysis and hydrogen exchange experiments.^37,38^ We show here that the aggregation propensity of this region is conferred, at least in part, by the unusually high content of aromatic residues in this sequence, such that removal of a single aromatic moiety or the introduction of a single charge in this region has a dramatic effect on fibril growth. Interestingly, the crystal structure of β_2_m in the major histocompatibility complex (MHC) class I complex illustrates that aromatic residues in this region (Phe56, Trp60, Phe62 and Tyr63) form important interactions with the α_1_α_2_ domains of the MHC heavy chain, while Leu65 makes contact with the α_3_ domain, suggesting a functional requirement for the evolution of this sequence, despite its high amyloid potential.^39^ The functional significance of these residues is highlighted by the fact that they are all highly conserved across β_2_m sequences from many species, whilst other aromatic amino acids found in this region of human β_2_m (Y66 and Y67) are not retained in other sequences.^40,41^ In the C_H_3 domain of the Fc fragment (A342–A443) that belongs to the same structural class of immunoglobulin domains as β_2_m, the equivalent region of the sequence differs markedly (GSFFLYSKLTVD and WSFYLLYYTEFT, respectively; identical residues are underlined).^42^ Although human β_2_m contains this aggregation-prone sequence, it appears to have developed mechanisms to avoid aggregation by burying this region in the core of the MHC complex and, for the native monomeric protein, by sequestering this strand within the centre of the β-sheet involving strands A, B, E and D, such that its full aggregation potential is not revealed.^9,43^ Indeed, natively folded β_2_m can be incubated for extended periods of time at millimolar concentrations (> 1000 times higher than the *in vivo* concentration) in the absence of seeds without detectable amyloid formation.^18,44^ This therefore explains why amyloid formation can occur under conditions that encourage unfolding,^14,15,45,46^ whereupon residues 62–70 may become exposed and realise their fibrillogenic potential.

### Mechanisms of β_2_m aggregation

**Fig. 7:**
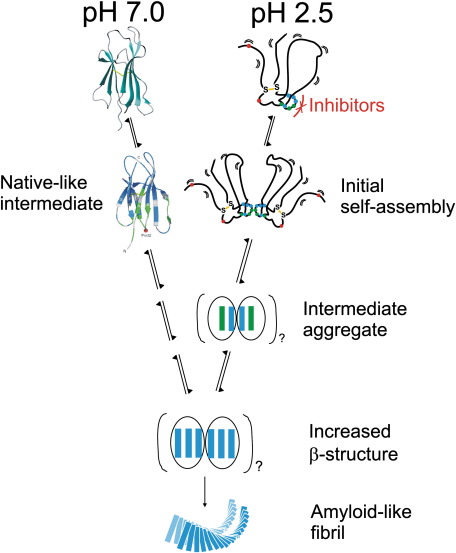
Schematic diagram of a possible mechanism for β_2_m aggregation at pH 7.0 and pH 2.5. Aggregation at pH 7.0 proceeds *via* a native-like intermediate.^44^ At pH 2.5, β_2_m is highly unfolded, with the residues in the region 62–70 participating in a non-native hydrophobic cluster. This is shown in the figure by circles coloured as in Fig. 5. A single disulphide bond (yellow) links residues 25–80 and is essential for fibril formation.^12^ In the initial stages of fibril formation, we propose that residues 62–70 form one key complementing surface involved in both the nucleation and the elongation phases of fibril growth and may provide a target for therapeutic molecules to disrupt amyloid formation. Since the fibrils formed from a native-like state at neutral pH and those formed from an acid-denatured state are morphologically indistinguishable,^47^ the fibril formation pathways presumably must converge, although the point at which they do so remains uncertain. The precise details of the size of the nucleus and the structure of the fibrils remain unresolved.

It has previously been observed that β_2_m forms amyloid-like fibrils at pH 7 *via* an on-pathway folding intermediate (Fig. 7).^44^ This aggregation-prone species possesses a highly native-like structure according to chemical shift analysis of NMR spectra, but contains a *trans* His31–Pro32 peptide bond compared with the *cis* isomer found in native β_2_m.^39^ Despite analysis of this early aggregation-prone species, the mechanism of further assembly steps remains elusive. Interestingly, a key role for the region encompassing residues 62–70 in the aggregation of β_2_m at acidic pH is consistent with the increased amyloidogenicity observed under physiological conditions for the derivative containing a single cleavage of the polypeptide chain at Lys58.^48^ In this variant, the aromatic-rich region of the protein involving residues 62–70 may be less conformationally constrained, increasing the opportunity for it to participate in intermolecular interactions. How the aggregation mechanism under acidic conditions relates to that under physiological conditions is currently unresolved, and further experiments will be needed to determine directly the role of residues 62–70 in this pathway. Interestingly, there are a large number of amino acids bearing carboxyl groups within the 58–79 region (Asp59, Glu69, Glu74, Asp76 and Glu77) that, whilst most likely neutral under the acidic conditions of this study, are presumably charged under physiological conditions. Thus, electrostatic repulsion of these side chains may account for the slower aggregation rates of β_2_m at neutral pH.

The data presented here suggest that amyloid formation from acid-unfolded β_2_m involves both sequence and structural specificities, with individual residues in the region 62–70 playing different roles that depend not only on their physicochemical properties but also on their location in the sequence and on the structure of the self-assembling species. Based on these data, we suggest that the initial steps of β_2_m self-assembly into amyloid at pH 2.5 involve the association of two or more hydrophobically collapsed species, which present a specific surface involving residues 62–70 that, by an unknown mechanism, favours the transition of the unfolded protein into elongation-competent species (Fig. 7). This possibility is supported by mass spectrometric evidence, which indicates that β_2_m forms amyloid-like fibrils *via* small oligomeric species at low pH.^49^

The variants analysed here indicate that the region corresponding to the native β-strand E plays an important role in the nucleation and elongation of amyloid-like fibrils from β_2_m under acidic conditions *in vitro*. Determining whether other regions of the polypeptide chain are involved in these events—such as the B/C loop containing Phe30 that was predicted by Kozhukh *et al.*^50^ to be key to amyloid formation, or residues 83–89 shown by Ivanova *et al.*^40^ to be important in fibril formation based on a sequence comparison of human and mouse β_2_m—will require further detailed mutagenesis studies to resolve. The approach taken here, in which reproducible assays for analysing the rates of nucleation and elongation have been developed, lays the foundation for such studies. Perhaps most important are questions involving the nature of the nucleation- and elongation-competent species, including the number of aggregation-promoting regions, their structural organisation and their size. The data presented here suggest that similar regions of the protein are involved in both stages of aggregation for β_2_m, as has been observed previously for other proteins.^34^ Whether this is a generic characteristic of amyloid formation across protein systems and different growth conditions remains to be resolved.

Despite progress in understanding the mechanisms of amyloid formation, small-molecule therapies for this class of disorder still have to be developed, especially for proteins that lack an active site that can readily be targeted for inhibitor design.^51^ One approach to the development of therapeutic agents is the use of small molecules that specifically and efficiently inhibit the aggregation process. Indeed, a range of polyphenol molecules have recently been demonstrated to be capable of inhibiting the formation of fibrillar aggregates for targets such as Aβ, α-synuclein, Tau, islet amyloid polypeptide, insulin, calcitonin and prion protein.^52^ Interestingly, it appears that the structural properties of these small molecules contribute to their inhibitory effect, and it has been suggested that their ability to inhibit aggregation can be attributed to their aromatic character, which enables interactions with the amyloidogenic sequence.^52,53^ Such molecules may therefore be useful for inhibiting the aggregation of acid-unfolded β_2_m, based on our observation that the aromatic-rich portion of the β_2_m sequence is important in fibril formation. The fact that at least one distinct region of β_2_m is key to amyloid formation makes this part of the sequence a possible target for designing aggregation inhibitors (Fig. 7).^54^ Previously, short sequence stretches of proteins have been targeted by self-recognition peptidomimetic compounds that disrupt amyloid formation.^55^ For instance, many groups have used the structure-based introduction of *N*-methylated peptides for proteins such as Aβ, α-synuclein and islet amyloid polypeptide, which bind specifically at self-recognition sites to develop inhibitors of amyloid formation for these proteins.^56–58^ Other peptide-based inhibitors that are sequence-specific but inhibit aggregation by alternative mechanisms such as sequence alteration, insertion of β-breaker residues and terminal modifications have been described.^55^ Ultimately, the first stage of rationally designing such an inhibitor must be target identification. At least for acid-unfolded β_2_m, one such target is the aromatic-rich region comprising residues 62–70 identified here. The production of successful inhibitory molecules based on this target region may not only prove useful for further dissection of the mechanism of fibril assembly at pH 2.5 and may also provide useful tools for the delineation of the mechanism of fibril formation at neutral pH and of the stages, if any, at which the fibril formation mechanisms at acidic and neutral pH converge.

## Materials and Methods

### Materials

The *Escherichia coli* strain BL21(DE3) pLysS was obtained from Promega. Q-Sepharose and all other reagents were purchased from the Sigma-Aldrich Chemical Company. Spectrapore membrane (molecular mass cutoff, 3500 Da) was obtained from Spectrum Laboratories, Inc. Superdex 75 was purchased from Amersham Biosciences. Carbenicillin was obtained from Melford Chemicals. Deuterated solvents were obtained from Fluorochem Ltd. Oligonucleotides were purchased from MWG Biotech.

### Mutagenesis and protein purification

Mutagenesis was carried out using the QuikChange site-directed kit (Stratagene) with the plasmid pINK_WT_ as template.^14^ The recombinant human β_2_m variants were expressed in ^15^N-enriched minimal media and purified as described.^21^ The proteins were shown to be > 95% pure by SDS-PAGE and > 95% labelled with ^15^N, as determined using electrospray ionisation mass spectrometry. The proteins were stored at − 20 °C as lyophilised powder. Protein concentrations were calculated from *A*_280_ of the denatured state using the coefficients determined by the method of Gill and von Hippel.^59^

### Kinetic analysis of fibril growth

Data were recorded using a BMG FLUOstar Optima reader (BMG) and 96-well black-wall plates (Costar) sealed with clear sealing film (Axygen). Samples had a volume of 100 μl containing 100 μM ThT in 25 mM sodium acetate/25 mM sodium phosphate (pH 2.5) and 0.04% (wt/vol) NaN_3_. The proteins were all dissolved at approximately twice the final required concentration as stock solutions in ∼ 5 mM HCl and centrifuged at ∼ 400,000*g* for 30 min at 10 °C. Only the upper third of the solution was used for fibril growth experiments to ensure that each reaction commenced with soluble protein. These samples were confirmed to contain only monomeric material by analytical gel filtration and analytical ultracentrifugation. Protein was added to each well such that the final concentration was 89 μM for each variant. Measurements were acquired simultaneously for six replicates of each variant. The plate was incubated at 25 °C with orbital shaking (1 mm width, 600 rpm), and data points were recorded every 5 min, with the fluorescent measurements representing the average from 20 flashes. To follow ThT fluorescence, the samples were excited at 440 nm, and the fluorescence emission was measured at 480 nm. In the case of seeded growth, the same protocol was followed, except that 2% (vol/vol) seed was added to the initial reaction. The seed consisted of wild-type β_2_m fibrils, formed previously at pH 2.5, that had been fragmented by four freeze–thaw cycles and stored at − 80 °C in multiple aliquots. This ensured that the same seeds were used for all reactions. The fibrils used as seeds were grown using wild-type β_2_m (89 μM) in 25 mM sodium acetate/25 mM sodium phosphate (pH 2.5) and 0.04% (wt/vol) NaN_3_ at 37 °C with shaking for 14 days and were determined to be long, straight amyloid-like fibrils by negative-stain electron microscopy (EM). To obtain an independent verification of fibril growth, test reactions were performed using conditions identical to those described above, but the progress of fibril growth was measured by the change in ThT fluorescence and by intrinsic tryptophan fluorescence (with excitation at 280 nm and with emission measured at 350 nm). Both methods showed identical kinetics, where an increase in ThT fluorescence is associated with a concomitant decrease in intrinsic tryptophan fluorescence.

The observed growth traces showed differences in the final ThT fluorescence signal for the different variants, yet usually the same final fibril yield (determined by absorbance measurements of soluble material after centrifugation) and fibril morphology. Therefore, all the data were normalised by assuming a final ThT signal of 100%, and the resulting curves were used to determine the lag times and apparent rate of elongation. The lag time was obtained by fitting a straight line to the initial slope of the growth phase. The time at which this line intersected with the baseline was taken as the lag time.^60^ Fits of the apparent elongation rates were carried out by using only the part of the curve corresponding to the growth phase and by assuming exponential growth with the formula *y* = *A* + *B*e^(−*kx*)^. The value of *k* was taken as the apparent rate of elongation. In the case of seeded growth, the inverse of the time taken to reach 50% of the final normalised ThT signal was used as an indication of the apparent rate of elongation.

### NMR sample preparation

All NMR experiments were carried out using 5 mg/ml ^15^N-labelled β_2_m in 90% (vol/vol) H_2_O/10% (vol/vol) ^2^H_2_O at pH 2.5. The pH was adjusted using dilute HCl.

### NMR spectroscopy

NMR experiments were performed at 25 °C using a Varian Unity Inova spectrometer operating at a ^1^H frequency of 500 MHz. Gradient-enhanced ^1^H–^15^N HSQC spectra were acquired using 128 complex points and 16 scans per increment, with spectral widths of 4508 Hz and 1200 Hz in the ^1^H and ^15^N dimensions, respectively. Watergate solvent suppression was used, and all NMR data were processed using NMRPipe.^61^ The data were apodised using a cosine bell function, followed by zero filling and Fourier transform. The two-dimensional spectra were analysed in NMRView.^62^

Backbone ^15^N transverse relaxation measurements [transverse relaxation rate, *R*_2_ = (*T*_2_)^− 1^] were carried out as described.^63^ Duplicate points and the spectral noise levels were used to obtain an estimate of the error. The *R*_2_ relaxation measurements of wild-type β_2_m and the described variants were performed at 500 MHz at pH 2.5 in dilute HCl at 25 °C using a series of 11 experiments, with mixing times ranging from 16.32 ms to 456.96 ms. The transverse relaxation data for all of the experiments at pH 2.5 were analysed using models for increasing complexity, as described previously.^21,64,65^
